# Supplementary material for: Comparative transcriptomics reveals differences in cortical cell type organization between metatherian and eutherian mammals
Source: PNAS Nexus. 2026 Apr 16;5(4):pgag055. doi: 10.1093/pnasnexus/pgag055 (PMC13089494; doi:10.1093/pnasnexus/pgag055)
Supplement: pgag055_Supplementary_Data [file pgag055_supplementary_data.pdf]

Comparative transcriptomics reveals differences in cortical cell type organization between metatherian and eutherian mammals

Authors: Ryan Gorzek and Joshua T. Trachtenberg

Affiliations: Department of Neurobiology, David Geffen School of Medicine, University of California, Los Angeles; Los Angeles, CA 90095, USA

Corresponding author: Joshua Trachtenberg, joshua.trachtenberg@gmail.com

Author Contributions: Conceptualization: JTT; Resources: JTT; Data curation: RG; Software: RG; Formal Analysis: RG, JTT; Validation: RG; Investigation: RG, JTT; Visualization: RG; Methodology: RG; Supervision: JTT; Project Administration: JTT; Funding Acquisition: JTT; Writing – Original Draft: RG, JTT; Writing – Review and Editing: RG, JTT.

Competing Interest Statement: Joshua Trachtenberg is the co-owner of Neurolabware, LLC.

Classification: Biological Science; Neuroscience

Keywords: Neocortex, transcriptomics, comparative, evolution, column

This file includes:

Supplementary Figures 1 to 4
Supplementary Table

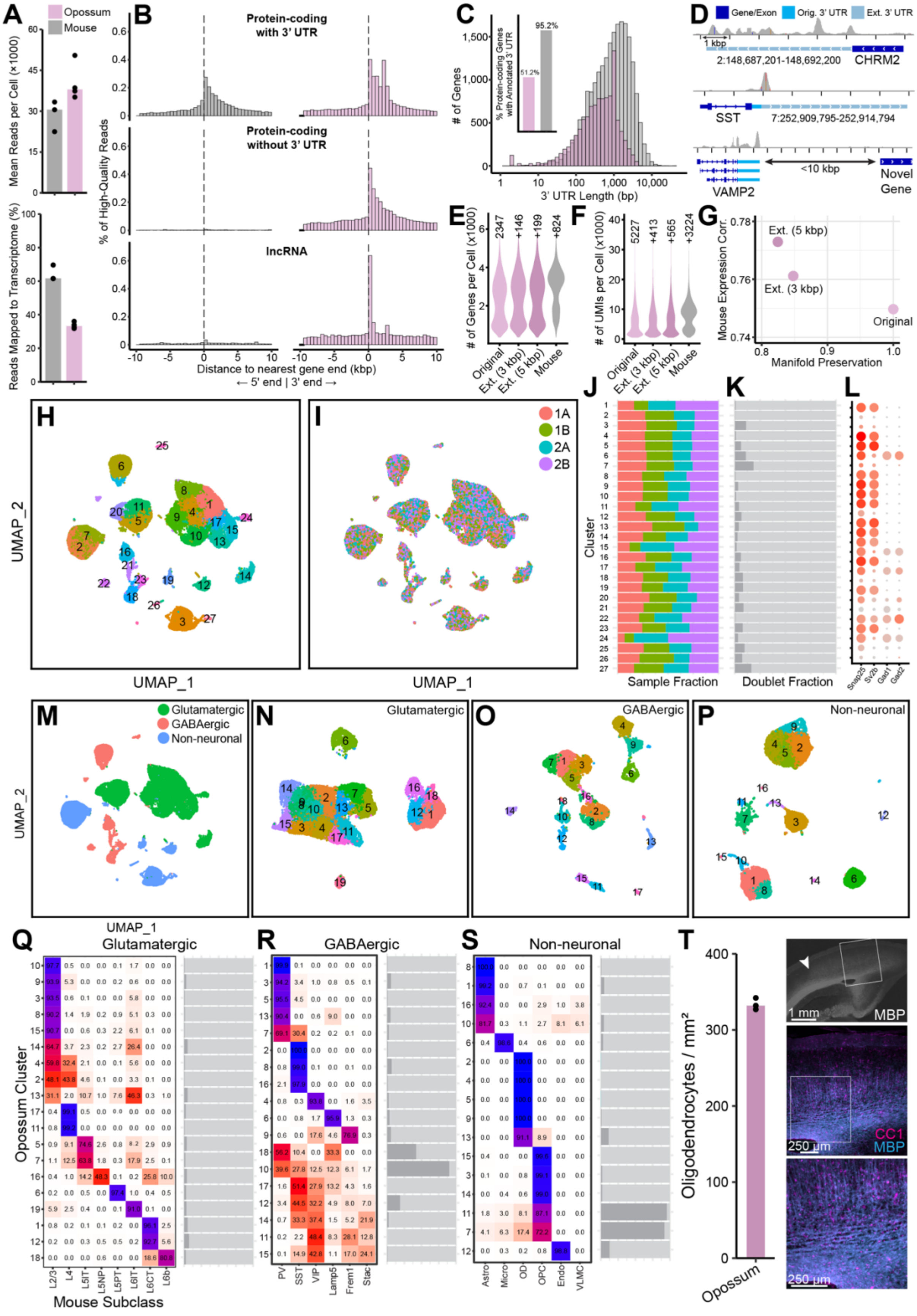

A
Opossum
Mouse
Mean Reads per Cell (×1000)
Reads Mapped to Transcriptome (%)
B
Protein-coding with 3' UTR
Protein-coding without 3' UTR
lncRNA
% of High-Quality Reads
Distance to nearest gene end (kbp)
← 5' end | 3' end →
C
# of Genes
% Protein-coding Genes with Annotated 3' UTR
51.2%
95.2%
3' UTR Length (bp)
D
Gene/Exon
Orig. 3' UTR
Ext. 3' UTR
1 kbp
2:148,687,201-148,692,200
CHRM2
SST
7:252,909,795-252,914,794
<10 kbp
VAMP2
Novel Gene
E
# of Genes per Cell (x1000)
2347
+146
+199
+824
Original
Ext. (3 kbp)
Ext. (5 kbp)
Mouse
F
# of UMIs per Cell (x1000)
5227
+413
+565
+3224
G
Mouse Expression Corr.
Manifold Preservation
Ext. (5 kbp)
Ext. (3 kbp)
Original
H
I
1A
1B
2A
2B
UMAP_1
UMAP_2
J
Cluster
Sample Fraction
K
Doublet Fraction
L
Snap25
Sv2b
Gad1
Gad2
M
Glutamatergic
GABAergic
Non-neuronal
N
Glutamatergic
O
GABAergic
P
Non-neuronal
Q
Glutamatergic
Opossum Cluster
Mouse Subclass
L2/3
L4
L5IT
L5NP
L5PT
L6IT
L6CT
L6b
R
GABAergic
PV
SST
VIP
Lamp5
Frem1
Stac
S
Non-neuronal
Astro
Micro
OD
OPC
Endo
VLMC
T
Oligodendrocytes / mm²
Opossum
1 mm
MBP
250 µm
CC1
MBP
250 µm

Figure S1. Opossum snRNA-seq data preprocessing.

(**A**) Despite comparable sequencing depths, the percentage of snRNA-seq reads mapping confidently to the transcriptome (exons, introns, and untranslated regions [UTRs]) in opossums was nearly half of that in mice. (**B**) Intergenic (mapping confidently to regions outside of exons, introns, or UTRs) read locations relative to the closest gene (negative is 5' or upstream, positive is 3' or downstream) in mice and opossums. Distributions are shown by gene biotype (protein-coding with or without an annotated 3' UTR, or long noncoding RNA [lncRNA]) of the closest gene, as a fraction of total high-quality reads. Because most mouse protein-coding genes have an annotated 3' UTR (see panel C), most intergenic reads fall into that group. Note the bimodality of that group in opossums (related to panel C). (**C**) Fraction of genes with an annotated 3' UTR in both species, and distribution of their lengths. Note small spikes at 3 bp (stop codons) and 1000 bp (consistent with annotation artifacts). (**D**) Examples of gene extension that capture downstream read pileups in opossums, even when 3' UTR annotation is already present (e.g., SST). Genes less than 10 kbp upstream of a neighboring gene were not extended (e.g., VAMP2). (**E–F**) Distribution of genes (E) and transcripts (UMIs; F) per cell in original and extended (3 or 5 kbp) opossum genome configurations, relative to mice. The median is shown for the original opossum genome, with others shown as relative increases. (**G**) Manifold preservation plotted against mouse expression correlation for opossum genome configurations. Manifold preservation was quantified as the mean fraction of each cell's k=30 nearest neighbors in PCA space (30 PCs) that are shared between the original and extended genomes: a value of 1.0 indicates identical local structure. Mouse expression correlation was computed as the mean Spearman correlation coefficient between opossum and mouse pseudobulk expression profiles aggregated by cell class (glutamatergic, GABAergic, non-neuronal). (**H**) Unsupervised Leiden clustering of all opossum cells passing initial quality control (see Methods) in UMAP space. (**I–J**) UMAP (I) and cluster-wise (J) representations of samples in opossum snRNA-seq data. Numbers (1 and 2) represent biological replicates and letters (A and B) represent technical replicates. (**K**) Cluster-wise representations of putative doublets (see Methods). (**L**) Canonical neuronal (*Snap25*), glutamatergic (*Sv2b*), and GABAergic (*Gad1*, *Gad2*) marker genes used to divide unsupervised clusters into cell classes. (**M**) All opossum cells divided into cell classes. (**N–P**) Unsupervised clustering of glutamatergic (N), GABAergic (O), and non-neuronal (P) cells prior to removal of ambiguous or doublet-enriched clusters. (**Q–S**) Cluster-wise cross-species correspondence (left) and doublet representation (right) for glutamatergic (Q), GABAergic (R), and non-neuronal (S) classes used to guide removal of ambiguous/artifact clusters. (**T**) Oligodendrocyte densities in opossum V1 (left) were measured in sections co-labeled for CC1 (mature oligodendrocytes; magenta) and myelin basic protein (MBP; cyan) marking myelinated processes (right). Related to Fig. 1K, see also (*42*).

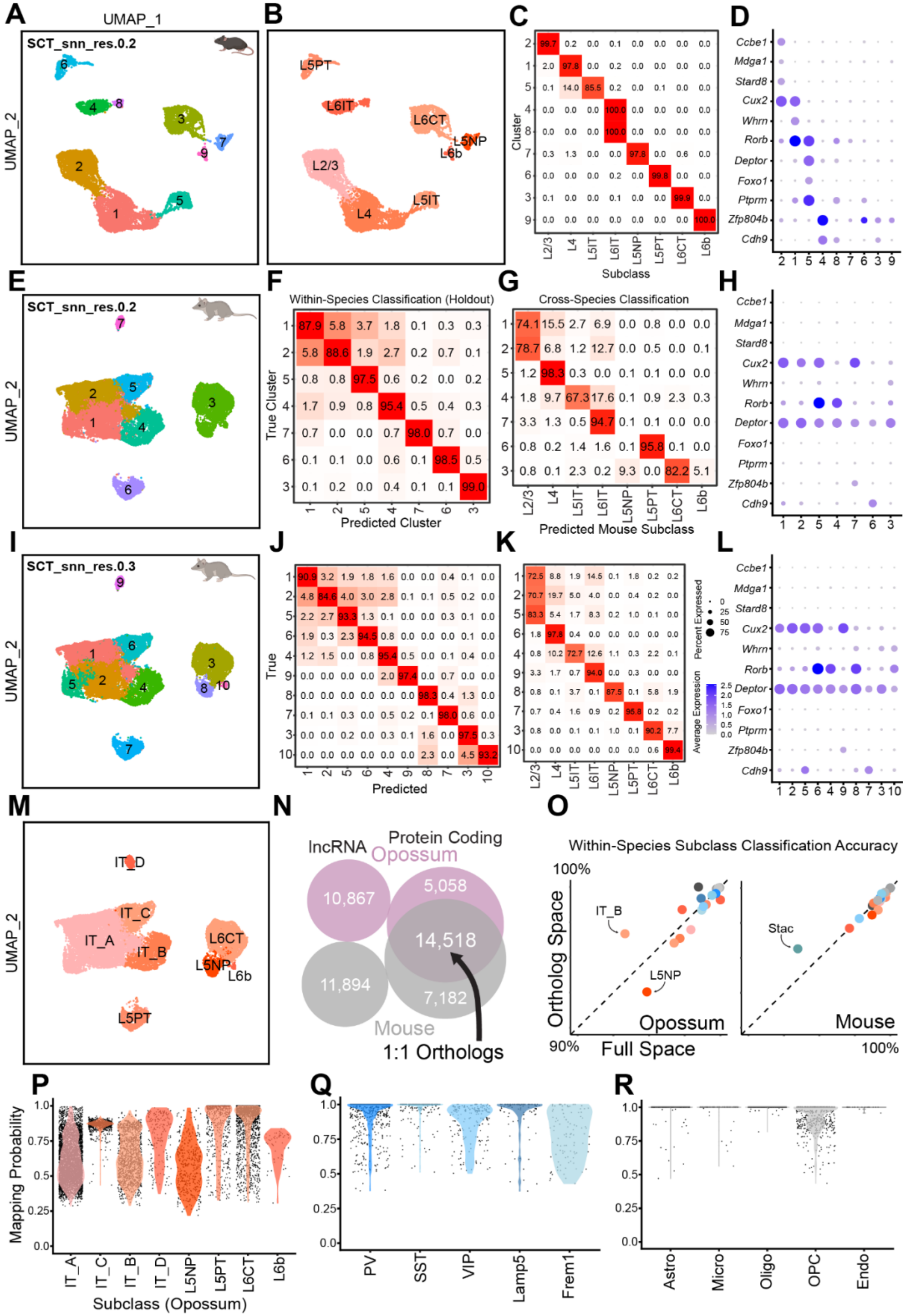
A
B
C
D
UMAP_1
UMAP_2
SCT_snn_res.0.2
L5PT
L6IT
L6CT
L5NP
L6b
L2/3
L4
L5IT
Cluster
Subclass
Ccbe1
Mdga1
Stard8
Cux2
Whrn
Rorb
Deptor
Foxo1
Ptprm
Zfp804b
Cdh9
E
F
G
H
Within-Species Classification (Holdout)
True Cluster
Predicted Cluster
Cross-Species Classification
Predicted Mouse Subclass
I
J
K
L
SCT_snn_res.0.3
True
Predicted
Percent Expressed
Average Expression
M
N
O
IT_A
IT_B
IT_C
IT_D
lncRNA
Protein Coding
Opossum
Mouse
1:1 Orthologs
10,867
5,058
14,518
11,894
7,182
Within-Species Subclass Classification Accuracy
100%
90%
Ortholog Space
Full Space
Stac
P
Q
R
Mapping Probability
Subclass (Opossum)
PV
SST
VIP
Lamp5
Frem1
Astro
Micro
Oligo
OPC
Endo

Figure S2. Semi-supervised labeling of opossum intratelencephalic (IT) subclasses and cross-species correspondence.

(**A**) Mouse glutamatergic cells in UMAP space, clustered at low resolution (0.2) with the Leiden algorithm. (**B**) Mouse glutamatergic cells in UMAP space, labeled by subclass (*30*). (**C**) Confusion matrix between low-resolution unsupervised clustering and subclass labels in mice. (**D**) Canonical glutamatergic subclass marker gene expression in unsupervised clusters shown in (A). (**E**) Opossum glutamatergic cells in UMAP space, clustered at low resolution (0.2). (**F**) Confusion matrix derived from 50% train-test splits of unsupervised clusters shown in (E). (**G**) Confusion matrix derived from cross-species label transfer (see Methods) between mouse glutamatergic subclasses and unsupervised clusters shown in (E). (**H**) Canonical glutamatergic subclass marker gene expression in unsupervised clusters shown in (E). (**I–L**) Same as (E–H), but with Leiden resolution set to 0.3. (**M**) Final glutamatergic subclass assignment in opossums. Clusters 1 and 2 in (E) were merged to form IT_A. L6CT, L5NP, and L6b correspond to clusters 3, 8, and 10 in (I). (**N**) Composition of the opossum genome. Long noncoding RNAs (lncRNAs) are species-specific and one-to-one orthologs are exclusively protein-coding genes. (**O**) Classification accuracy of 50% train-test splits for each subclass in species-specific (full) and orthologous gene expression space in opossums and mice. (**P–R**) Cross-species mapping quality (obtained from MapQuery, see Methods) scores for opossum cells shown by glutamatergic (P), GABAergic (Q), and non-neuronal (R) subclasses. Related to Fig. 2C–F.

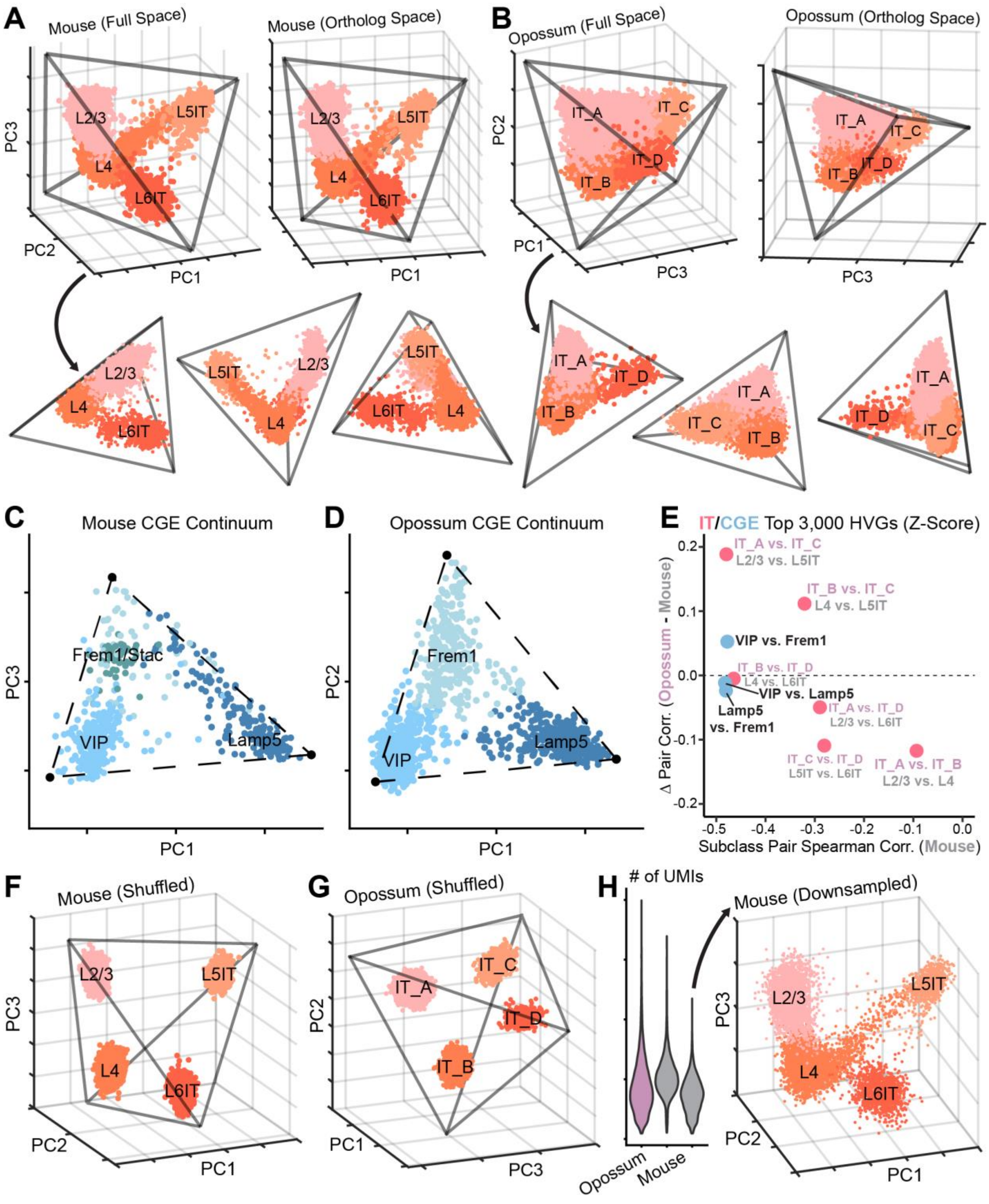

Figure S3. IT principal component (PC) spaces, other gradients, and quality controls.

(**A–B**) Tetrahedral IT gradients in mouse (A) and opossum (B) principal component spaces, shown from various angles. Tetrahedrons were fit using established algorithms (see Methods).

Related to Fig. 2G–H. (**C–D**) GABAergic neurons derived from the caudal ganglionic eminence (CGE), which form a continuum in gene expression space, display similar triangular gradients in mice (C) and opossums (D). (**E**) Relationship between within-species subclass similarity and cross-species divergence for IT and CGE continua. Each point represents a pair of IT or CGE subclasses. The x-axis shows the Spearman correlation of subclass-pair gene expression in mice, computed over the top 3,000 highly variable genes (z-scored within IT or CGE subclasses). The y-axis shows the difference in this pairwise correlation between opossum and mouse (opossum − mouse). Positive values indicate increased similarity in opossum relative to mouse, while negative values indicate reduced similarity. Note that CGE subclass pairs have similar within-continua similarity to L2/3 and L5IT, but this correlation (IT_A vs. IT_C) increases in opossums considerably more than the CGE pairs. (**F–G**) Randomly shuffling gene expression within subclasses (see Methods) confirms that mouse (F) and opossum (G) IT cells form continua that are not driven by noise (see Methods). (**H**) The mouse IT continuum retains its structure after downsampling UMI (transcript) counts to match opossum levels (see Methods).

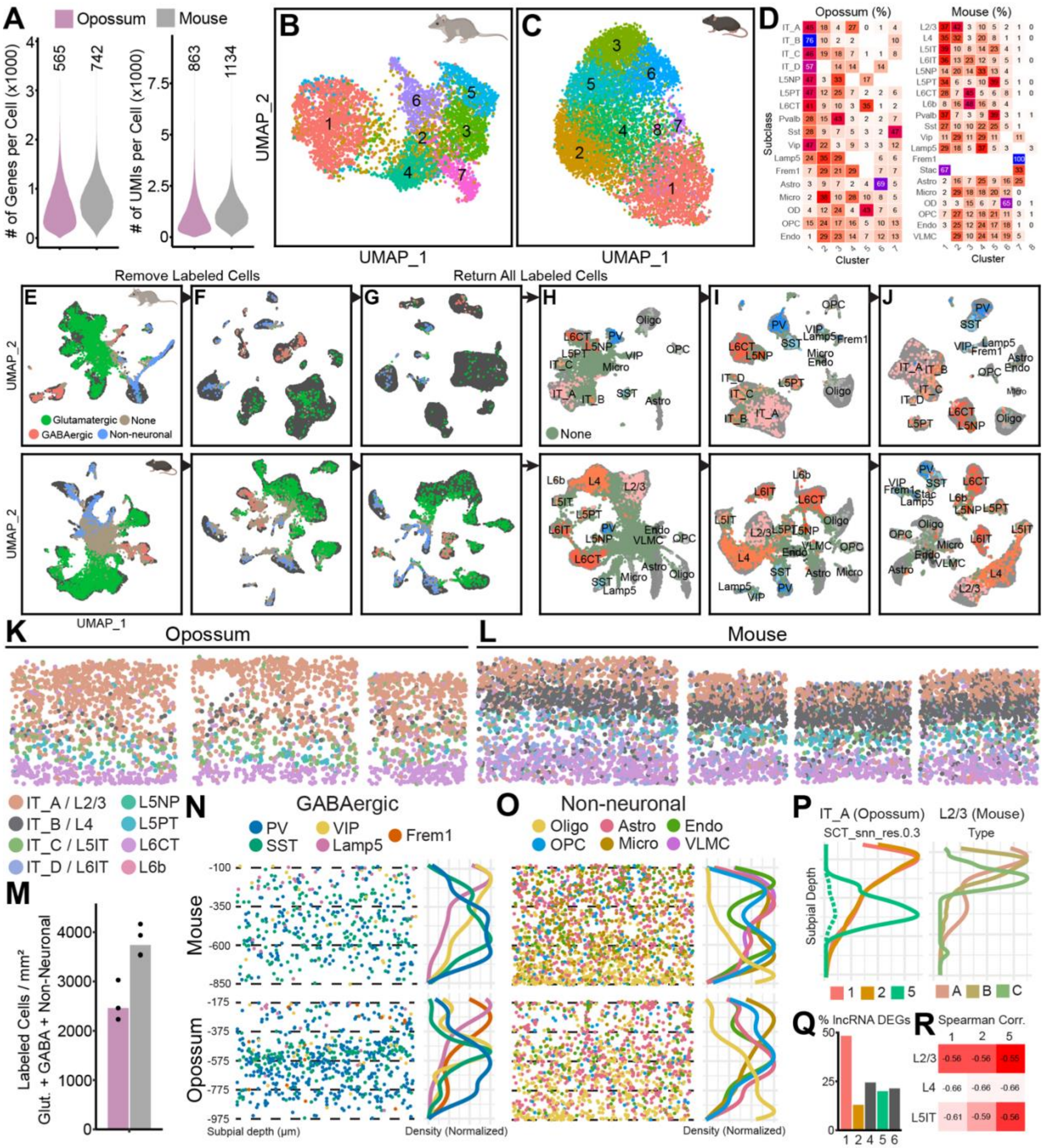

Figure S4. Stereo-seq preprocessing and non-glutamatergic cell subclass distributions.

(**A**) Number of genes (left) and UMIs (right) per cell in all Stereo-seq cells from mice and opossums. (**B–C**) Unsupervised clustering of opossum (B) and mouse (C) Stereo-seq cells. (**D**) Final subclass (the result of H–I, see Methods) membership among unsupervised clusters in the original Stereo-seq space (B–C) for opossums (left) and mice (right). (**E–G**) UMAP representation of integrated snRNA-seq (gray) and Stereo-seq (colored by class) cells in opossums (top) and mice (bottom). Stereo-seq class labels were assigned using nearest

neighbors in PCA space (see Methods). (**H–J**) Same as (E–G), but with Stereo-seq cells labeled by subclass. Ambiguous cells labeled ‘None’ in (G) were removed before the integration step in (H) (see Methods). (**K–L**) Individual cortical columns extracted from opossum (K) and mouse (L) Stereo-seq sections. Only glutamatergic cells are shown, colored by subclass. (**M**) Total cell density (cells per mm^2) for mouse and opossum Stereo-seq data following class assignment (see Methods). (**N–O**) Distribution of non-neuronal (N) and GABAergic (O) subclasses from pooled V1 regions. Related to Fig. 3C–D. (**P**) Spatial distributions of IT_A subclusters at Leiden resolution 0.3 (left; fig. S2I–L) and mouse L2/3 types (right). The dashed line for subcluster 5 is normalized to the peak of cluster 1. (**Q**) Fraction of IT subcluster DEGs that are long non-coding RNAs (lncRNAs) at Leiden resolution 0.3 (fig. S2I–L). See Methods for further discussion. (**R**) Spearman correlation between the top 3,000 highly variable genes (HVGs) of opossum IT_A subclusters (Leiden resolution 0.3) and mouse IT subclasses. See Methods for further discussion.

Figure	Panel	Test	Statistic	P-Value
1	G	ANOVA (fraction ~ species * cell_class)	F = 19.74 (species:cell_class)	0.00125
		Tukey MC t-Test (Mouse:Glut. – Opossum:Glut.)		0.04385
		Tukey MC t-Test (Mouse:GABA. – Opossum:GABA.)		0.04385
	H	ANOVA (fraction ~ species * cell_subclass)	F = 0.917 (species:cell_subclass)	0.47000
	I	ANOVA (fraction ~ species * cell_origin)	F = 48.46 (species:cell_origin)	0.00004
		Tukey MC t-Test (Mouse:MGE – Opossum:MGE)		0.00281
		Tukey MC t-Test (Mouse:CGE – Opossum:CGE)		0.00281
	J	ANOVA (fraction ~ species * cell_subclass)	F = 47.93 (species:cell_subclass)	2.07e-13
		Tukey MC t-Test (Mouse:PV – Opossum:PV)		0.00000
		Tukey MC t-Test (Mouse:SST – Opossum:SST)		0.00021
		Tukey MC t-Test (Mouse:VIP – Opossum:VIP)		0.00411
		Tukey MC t-Test (Mouse:Lamp5 – Opossum:Lamp5)		0.10668
		Tukey MC t-Test (Mouse:Frem1 – Opossum:Frem1)		0.10076
	K	ANOVA (fraction ~ species * cell_subclass)	F = 92.14 (species:cell_subclass)	2.0e-16
		Tukey MC t-Test (Mouse:Astro – Opossum:Astro)		0.01812
		Tukey MC t-Test (Mouse:Micro – Opossum:Micro)		0.00011
		Tukey MC t-Test (Mouse:Oligo – Opossum:Oligo)		0.00000
		Tukey MC t-Test (Mouse:OPC – Opossum:OPC)		0.40149
		Tukey MC t-Test (Mouse:Endo – Opossum:Endo)		1.8e-06
S1	T	ANOVA (fraction ~ species * cell_subclass)	F = 70.95 (species:cell_subclass)	7.8e-11
		Tukey MC t-Test (Mouse:Astro – Opossum:Astro)		0.00013
		Tukey MC t-Test (Mouse:Micro – Opossum:Micro)		0.00011
		Tukey MC t-Test (Mouse:Oligo – Opossum:Oligo)		0.00000
		Tukey MC t-Test (Mouse:OPC – Opossum:OPC)		0.99837
3	I	Wilcoxon Rank-Sum Test		0.0571
	J	Partial Spearman Perm. Test (n=10,000)	Mouse Vtx. B | C: r = -0.243	0.00000
		Partial Spearman Perm. Test (n=10,000)	Mouse Vtx. C | B: r = 0.086	0.0046
		Partial Spearman Perm. Test (n=10,000)	Opossum Vtx. B | C: r = -0.397	0.00000
		Partial Spearman Perm. Test (n=10,000)	Opossum Vtx. C | B: r = -0.116	0.00020
4	B	Wilcoxon Rank-Sum Test	W = 40	0.00822
	C	ANOVA (density ~ species * layer)	F = 135	2.0e-16
		Tukey MC t-Test (Mouse:L1 – Opossum:L1)		1.00000
		Tukey MC t-Test (Mouse:L2/3 – Opossum:L2/3)		0.33742
		Tukey MC t-Test (Mouse:L4 – Opossum:L4)		0.00021
		Tukey MC t-Test (Mouse:L5 – Opossum:L5)		0.00002
		Tukey MC t-Test (Mouse:L6 – Opossum:L6)		0.27145
	E	ANOVA (density ~ species * layer)	F = 5.387 (species:layer)	0.00216
		Tukey MC t-Test (Mouse:L1 – Opossum:L1)		0.98782
		Tukey MC t-Test (Mouse:L2/3 – Opossum:L2/3)		0.99904
		Tukey MC t-Test (Mouse:L4 – Opossum:L4)		0.99996
		Tukey MC t-Test (Mouse:L5 – Opossum:L5)		0.72384
		Tukey MC t-Test (Mouse:L6 – Opossum:L6)		0.00028

Table S1. Statistical tests, test statistics, and p-values for all main and supplementary figures.
